# Potential Risk to Brain Health after Surgical Interventions: Biomarkers to Predict the Occurrence of Cognitive Decline

**DOI:** 10.14336/AD.2025.1105

**Published:** 2026-10-18

**Authors:** Julio Rey-Picazo, Jimena Pita, Lucía Peña, Marta Ferraz-Torres, Oscar Martínez-García, Marina Ávila-Villanueva, Gloria Santos-Pérez, David López-Sanz, M. Victoria Moreno-Arribas, Carlos González Perrino, Vicente Muedra, Alfredo Jesús Miñano-Molina, Vicente Hernández-Rabaza, Ulises Gómez-Pinedo, Gonzalo León-Espinosa

**Affiliations:** ^1^Department of Anesthesiology, Institute of Neuroscience, Hospital Clínico San Carlos, Madrid, Spain;; ^2^Departamento de Química y Bioquímica, Facultad de Farmacia, Universidad San Pablo-CEU, CEU Universities, Madrid, Spain;; ^3^Navarra's Health Research Institute (IdiSNA), Navarra, Spain; ^4^Faculty of Health Sciences, Public University of Navarre, Pamplona, Spain; ^5^Faculty of Health Sciences, International University of La Rioja, Spain; ^6^University Hospital of Navarra, Pamplona, Navarra, Spain; ^7^Departamento de Psicología Social, del Trabajo y Diferencial, Facultad de Psicología. Universidad Complutense de Madrid, Spain;; ^8^Departament of Anesthesiology, Hospital Universitario Ramón y Cajal, Madrid, Spain;; ^9^Department of Experimental Psychology, Cognitive Processes and Speech Therapy, Complutense University of Madrid (UCM), Campus de Somosaguas, Pozuelo de Alarcón, Madrid, Spain;; ^10^Institute of Food Science Research (CIAL), CSIC-UAM, Campus de Cantoblanco, Madrid, Spain; ^11^Departamento de Cirugía, Facultad de Ciencias de la Salud, Universidad CEU Cardenal Herrera, Valencia, Spain;; ^12^Institut de Neurociències i Departament de Bioquímica i Biologia Molecular, Universitat Autònoma de Barcelona, Barcelona, Spain;; ^13^Centro de Investigación Biomédica en Red en Enfermedades Neurodegenerativas (CIBERNED), Madrid, Spain;; ^14^Unitat de Neurociència Traslacional, Parc Taulí Hospital Universitari, Institut d'Investigació i Innovació Parc Taulí (I3PT), Barcelona, Spain;; ^15^Department of Biomedical Sciences, Faculty of Health Sciences, Institute of Biomedical Sciences, Cardenal Herrera-CEU University, CEU Universities, Valencia, Spain; ^16^Laboratory of Neurobiology, Institute of Neurosciences, Hospital Clínico San Carlos Health Research Institute, Universidad Complutense de Madrid, Madrid, Spain.;

**Keywords:** Anesthesia, tau phosphorylation, dementia, Alzheimer’s disease;, neurodegeneration, surgery

## Abstract

As the global population ages, the number of people living with dementia is projected to increase significantly. Estimations indicate that over 150 million people worldwide will be living with dementia by 2050, with Alzheimer’s disease being the most common cause. Elderly people are also at greater risk of undergoing surgery, either elective or emergency, escalating the associated likelihood leading to cognitive decline, especially if accumulative. However, the relationship between surgery and dementia development remains controversial. The cause seems to lie in the heterogeneous preoperative state of subjects participating in research studies. Interpreting and comparing the results of these studies could be an arduous task due to variables such as medication, follow-up time, type of surgery and anesthesia, duration and invasiveness of the surgical intervention, differential neuroinflammatory response, the patient metabolic/biochemical status or if there are comorbidities. Considering the complexity of this type of studies, the present review summarizes the most important factors/biomarkers that could provide useful information for pre- and post-operative medical decision making in relation to the development of dementia. Emphasis will be placed on the relationship between temperature, Tau phosphorylation, whose plasma detection as an early diagnostic factor is gaining great relevance, and other neurodegenerative biomarker interplay. The prolonged maintenance of key biomarkers in blood could be detrimental and, therefore, a more comprehensive individualized hospital study may improve the prevention of postoperative complications.

## 1. Introduction

Given the large number of people undergoing surgery under general or locoregional anesthesia, it is a critical issue to investigate whether this exposure increases the risk of developing dementia and/or neurodegenerative diseases or accelerates the progression of an underlying cognitive disease. It has been reported that surgical interventions can cause postoperative delirium (POD), a relatively frequent cognitive impairment in elderly patients and/or in patients with neurocognitive disorders [1]. Although most clinical POD symptoms are reversible, recovery is usually slow and it may increase the risk of future dementia, especially in orthopaedical and cardiac surgeries [2,3,4]. Another related cognitive impairment that can occur after surgery is POCD (postoperative cognitive dysfunction), which is a temporary mental complication strongly linked to age [5,6]. Both POD and POCD can have significant negative consequences for the patient, such as increased morbidity, mortality, and hospital stay, and may even be a sign of preclinical Alzheimer's disease (AD), with a significant impact on socio-health resources [7]. AD is a multifactorial neurodegenerative disease, whose main risk factor is age and is the most common cause of dementia among older adults. There is no strong evidence to suggest that surgery directly increases the risk of developing AD [8], although some reports suggest that major surgery might accelerate memory decline in subjects who already have AD-related pathology [9]. Surgery is also associated with cerebral cortex thinning, which is a key characteristic in the pre-clinical stages of AD [10,11]. The preclinical stage of AD can last for several decades, offering an ideal point to initiate the treatment of the disease, before the onset of symptoms [12]. Age is undoubtedly the most influential factor leading to dementia risk, but surgery-associated neurotoxicity warrants further study, even in younger patients who may unknowingly be in that preclinical stage of AD.

## 2. Surgery and neuroinflammation

Surgical interventions trigger a systemic inflammation which may be the spark that ignites various subsequent brain complications: delirium, cognitive decline or neurocognitive disorders [13]. It is well documented that a wide variety of inflammatory mediators are released responding to injury, such as the Tumor Necrosis Factor alpha (TNF-α), nitric oxide (NO) or interleukins [14]. In chronic inflammatory conditions, interleukins, particularly IL-1β (secreted by monocytes and macrophages) and IL-17A (produced by T helper cells), may compromise the blood-brain barrier (BBB) integrity, allowing the monocyte-derived migration of macrophages into the brain. Inflammation is increasingly recognized as a key factor in the development and progression of AD, favouring Aβ deposition, diminishing the clearance of oligomeric tau proteins and accelerating tau spreading [15]. The neuroimmune system responds to this insult by activating a complex network of glial cells (microglia and astrocytes) and further releasing neuroinflammatory mediators and reactive oxygen and nitrogen species, which will increase the risk to develop dementia [16,17]. Notably, IL-6 and IL-8 have been highly correlated with long-term changes in cognitive function and are also increased with age and in AD [18,19].

Chronic inflammatory diseases, such as rheumatoid arthritis, are commonly treated with non-steroidal anti-inflammatory drugs (NSAIDs), which have a potential therapeutic use in reducing the risk of dementia [20,21,22]. NSAIDs (ibuprofen, naproxen or celecoxib, among others) are usually administered after surgery to reduce pain and inflammation. However, new formats of multimodal analgesia have been considered and the use of drugs that act through different mechanisms of action and routes of administration (pharmacological) is advocated. They can be used precisely before the start of surgery (inflammatory stimulation) to address pain before it occurs, and to reduce opioid use [23]. NSAIDs should be then correctly chosen and administrated, according to the type of surgery and patient and considering other factors such as efficacy, duration of action and dosage [24].

## 3. Cognitive dysfunction and Tau protein.

Blood biomarkers are currently promising tools for the prediction of cognitive impairment, dementia or neurodegenerative diseases. They are also increasingly utilized to monitor disease progression. Tau protein may be an accurate indicator of AD [25], as its hyperphosphorylation promotes abnormal aggregation and the consequent formation of neurofibrillary tangles (NFTs), a major histopathological hallmark in AD along with the senile plaques (formed by extracellular accumulations of β-amyloid peptide). Tau protein’s main function is the stabilization of cellular microtubules through the interaction with tubulin, but this interaction can be affected by post-translational modifications, capable of modifying the aggregation of Tau to form aberrant and toxic structures [26,27]. It has been reported that aberrant Tau protein induces a progressive neuronal loss and synaptic dysfunction, all of which may contribute to cognitive decline in AD [28].

Surgery, via the inflammatory response, might contribute to increase NFTs in the brain by a persistent activation of glial cells and the release of cytotoxic molecules [29]. However, the link between neuronal Tau aggregation and surgery is not fully demonstrated and the mechanism of Tau aggregation leading to the formation of NFTs in the brain remains unresolved. In this regard, the Tau proline-rich region has been described as a critical domain, where increased phosphorylation correlates with AD progression [30]. T181, T217 and T231 have recently gained great importance as biomarkers for early prediction of cognitive decline, even before its detection by PET (Positron Emission Tomography) scans [31,32]. pTau217 and pTau181 plasma detection are promising approaches for early AD diagnosis offering a non-invasive method to assess a comprehensive view of the pathology progression [33,34]. Recently, other Tau-based biomarkers (pS262 and pS356) have been suggested as reliable diagnostic candidates [35]. pTau217 and pTau181 plasma detection in people over 65 years of age are also useful to evaluate the POD risk [36]. Thus, phospho-Tau detection in blood is becoming an attractive target for the diagnosis of dementia-related pathologies in combination to other biological or clinical markers.

## 4. Hypothermia and Tau phosphorylation.

The elderly have the greatest problems regulating their body temperature, mainly due to less peripheral vasoconstriction and lower metabolic heat production. In this context, a common occurrence during surgery is perioperative hypothermia (when core temperature is below 36 °C) [37]. This decrease in temperature can be caused by a variety of reasons:

1. exposure to ambient temperature (the operating room is usually below 21°C).
2. patient's internal environment in contact with the cold external environment.
3. vasodilatation in locoregional anesthesia.
4. blood loss and/or infusion of intravenous fluids (which are at room temperature), especially when administered in large volumes.
5. decreased endogenous heat production because of anesthesia (thermoregulatory mechanisms are affected).
6. mechanical ventilation. Lack of humidification and heating of gas mixtures (oxygen/air/protoxide/ halogenated gases).

POCD is prevalent among elderly patients following both cardiac and orthopedic surgery, where glial hyperactivation or hyperphosphorylated Tau protein accumulation have been proposed as possible causes [38]. Regarding the potential relationship between temperature, surgery, and cognitive alterations, two types of surgery stand out above all:

### 4.1 Cardiac perioperative hypothermia

Surgical procedures for valve replacement/repair as well as interventions for aorto-coronary bypass (performed under extracorporeal circulation), require a controlled decrease in the patient's temperature (28-34ºC) as a traditional protective strategy. This mild hypothermia will decrease the cellular metabolism, which, in turn, will reduce brain damage and improve survival rates. It may also suppress the expression of pro-inflammatory cytokines, as have been demonstrated in cultured microglia following lipopolysaccharide stimulation [39]. However, other studies point out that there is still a lack of definitive evidence regarding the beneficial effects of mild hypothermia on clinical outcome [40].

Besides the role of hypothermia in brain health, other cardiac surgery factors have been linked to mental alterations. Patients with cardiac pathology usually have arterial hypertension, high cholesterol levels, diabetes or obesity, all of which are also associated with cognitive dysfunction and AD. In relation to this, a recent study described an association between antihypertensive use and atrial fibrillation with dementia risk [41]. Cardiac surgery also modifies total tau and pTau181 levels in blood, which may be associated with a higher risk of postoperative neurocognitive disorder or pNCD [42]. Unlike POD, which is acute, pNCD can last for even years following surgery.

In general, larger-scale longitudinal research is needed to further explore if cardiac surgery may accelerate neurodegenerative mechanisms.

### 4.2 Hip arthroplasty and hip osteosynthesis perioperative hypothermia

Cognitive complications after surgery are significantly associated to musculoskeletal surgeries, as described in a 9-year follow-up study [43]. The incidence is accumulative and increases in patients with two or more surgeries. The regional anaesthesia used in this type of surgery (spinal, epidural and peripheral nerve blocks) leads to vasodilation and heat loss (mild hypothermia). Although surgeons and anesthesiologists employ several actions to mitigate these effects, dementia is strongly associated with death in patients subjected to hip fracture surgery by a mechanism in which Tau phosphorylation may be participating [44,45]. A recent study confirmed that Aβ42, α-synuclein, and phospho-Tau, among others, might be associated with the occurrence rate of death in patients undergoing knee/hip arthroplasty [46]. None of these studies established a relationship between these outcomes and temperature variations, since temperature is not usually a systematically recorded variable in these surgeries.

### 4.3 Induced hypothermia in experimental animals

Animal research models are valuable tools for studying the relationship between Tau protein phosphorylation and temperature decrease, along with the long-term neurological impact this may have. It has been extensively tested that anesthesia promotes Tau protein phosphorylation through a decrease in brain temperature [47,48,49]. Subsequent studies in rats and mice confirmed that sevoflurane or propofol cause significant phosphorylation of Tau protein, accompanied by cognitive impairment [15]. Other types of anesthesia, widely used in experimental animals, such as isoflurane, also promote Tau phosphorylation, causing neuronal alterations and increasing the expression of AD-related genes [50]. Temperature drops in AD mouse models indicate that a reduced glucose metabolism aggravates AD prognosis [51]. To our knowledge, only one study suggests a relationship between lower body temperature and Tau phosphorylation in humans [52], noting that further studies are needed to demonstrate this association. However, Tau phosphorylation does not necessarily imply a negative neuronal effect, as Tau phosphorylation and dephosphorylation processes are reversible and physiologically relevant. For example, during early brain development, hibernation or hypothermia, Tau is phosphorylated in neurons at several residues [53,54]. In the case of hibernation, dephosphorylation occurs rapidly after the animal arouses and returns to euthermia [55]. It is hypothesized that this adaptive response is related to a neuroprotective mechanism. Elevated levels of pTau181 in the serum of the Syrian hamster during hibernation also disappear when the animal arouses [56]. This seems to indicate that pTau181 increase in the bloodstream is not associated with neurodegeneration (hibernation does not promote neuronal death), but probably with a temperature-decrease protection mechanism. In addition, although it is assumed that the origin of the Tau protein in blood is the brain (either by neuronal death or biomolecule content release across the blood brain barrier), it is not possible to state categorically, as Tau is also expressed in other tissues, such as skeletal muscle or kidney [57,58].

## 5. Sleep, anesthesia and Tau as three coordinate axes

There is ample evidence to support the beneficial effect of sleep on memory consolidation [59]. Some studies have suggested that sleep is beneficial for removing neurotoxic metabolites from the brain [60]. However, this statement is controversial as a recent study showed that brain clearance is even reduced during sleep and during the effect of anesthesia [61].

Emerging evidence suggests that sleep disruption is a potential risk factor for POD [62]. In addition, sleep and circadian rhythm disorders are also closely related to AD and mild cognitive impairment [63]. The hippocampus, which plays a crucial role in converting short-term memories into long-term memories, is one of the regions of the brain that is first affected in AD. Sleep dysfunction and AD also share abnormal temperature variations. Our body temperature naturally drops when we sleep (inhibited thermogenesis in NREM phase), a state where Tau protein may be hyperphosphorylated [64]. In contrast, sleep deprivation increases unphosphorylated Tau, while other changes take place at specific phosphorylation sites, with increasing levels of pT217 [65].

The use of animal models demonstrated that Tau protein plays a key role in sleep regulation. Tau knock-out mice showed a decrease in slow oscillations, sleep spindles and their coupling during the non-REM phase [66,67]. However, the overexpression of modified Tau (e.g., phosphorylated) alters the hippocampal theta activity in REM [68]. Although the exact role of brain temperature in AD and sleep is still under investigation, it has been proposed there is a correlation between Tau pathology, body temperature and sleep dysfunction aimed to accelerate the onset of cognitive decline [69,70].

Latest research increasingly implicates anesthesia as a potential contributor to cognition decline. The most used anesthetics in surgical interventions are sevoflurane (inhaled) and propofol (intravenous) and previous studies suggest adverse effects associated with their use, such as high glutamate levels, oxidative stress or even DNA damage. Specifically, sevoflurane has been shown to increase IL-6 levels, which could explain the cognitive impairment [71]. On the other hand, propofol is hypothesized to promote neuronal apoptosis through mitochondrial failure [72]. Despite this negative data, other clinical studies have not found a direct association between anesthesia exposure and neurocognitive deficits [73]. Both sevoflurane and propofol are used to induce and maintain general anesthesia, but in hip or knee replacement surgery, for example, regional anesthesia is also commonly used (spinal and epidural anesthesia). Regional anesthesia usually leads to a faster postoperative recovery, reduced nausea and decreased blood loss, and some studies reported a significant correlation between regional anesthesia and the decrease in POD incidence [74]. However, other studies found no evidence pointing to an effect on survival, cognitive decline or POD incidence compared with general anesthesia [75]. There is, therefore, controversy about the safety of anesthesia and side effects on cognitive function.

Anesthetics may also disrupt circadian rhythms [76] and sleep disturbance is a common occurrence in post-surgical patients [77]. The mechanisms are not well understood, although some studies propose microglial reactivity (M1 pro-inflammatory phenotype) as a key element [78]. Anesthesia and sleep are different states of altered consciousness, sharing several similarities at the biochemical level. For example, in both cases there is a reduction in the action of glutamate and an increase in the activity of gamma-aminobutyric acid (GABA), slowing down neuronal communication [79]. Cholinergic system alteration or loss of communication between the thalamus and the cerebral cortex are other shared overlapping characteristics [80].

It is noteworthy that melatonin administration has grown increasingly popular as a sleep aid, especially in elderly patients or in patients suffering sleep disorders. Melatonin is a hormone involved in the regulation of circadian rhythm and has been shown to improve cognitive function via Tau Phosphorylation reduction by downregulating GSK3β levels [81]. Thus, pharmacological medication should be considered when evaluating the effects of surgery on cognition.

## 6. Monitoring dementia after surgery using non-invasive techniques

Sustained systemic inflammation caused by surgery may result in long-term cognitive impairment, especially in vulnerable populations. Specific biomarker follow-up could be helpful to assess the risk of dementia developing or it could even serve to improve the individualized treatments administered after surgery [82,83]. Immunoassay-based biosensor (nanotechnology), Luminex bead-based array system, Proximity Extension™ Assay (Olink) or Simoa Planar Array Technology are recent advancements in ultra-sensitive quantification methods. Their great dynamic range, sensitivity and high precision are transforming early detection strategies, thus making it possible to accurately monitor different biomarkers over time with low invasiveness and low cost.

**Table 1:** Summary of plasma biomarkers that could provide useful information for pre- and post-operative medical decision making in relation to the development of dementia, emphasizing the pathophysiological mechanisms of the dual surgery-dementia role.

Biomarker (abbreviation)	Biomolecule description	Link to cognitive decline/surgery	Ref.
**Neurodegeneration and Pathological Aggregates**			
**Phosphorylated Tau protein (pTau)**	Phosphorylated Tau protein at sites T181, T217 and T231	Hyperphosphorylated Tau may cause aberrant aggregation and NFT formation. As discussed in previous sections, surgery and anesthesia can exacerbate Tau phosphorylation.	See section 3
**Amyloid beta** **(Aβ)**	The amyloid beta aberrant aggregation forms amyloid plaques in the brain of AD patients and is considered a core biomarker	Aβ42, Aβ40 and Aβ38 are obtained after the amyloid precursor protein (APP) is proteolyzed by secretases. Decreased CSF Aβ42 levels are observed in early AD, likely reflecting aggregation and deposition of Aβ into plaques in brain tissue and depleting levels in other fluids. Aβ42 has been associated to increase the delirium risk and the ratio Aβ42/40 may predict the onset and progression of AD. The ratio Aβ42/38 can also be used to assess early memory deficits.	[87,88]
**Neurofilament light chain (NfL)**	Neuronal protein highly expressed in myelinated axons	NfL high levels are indicative of axonal damage. Promising biomarkers to detect AD-associated cognitive decline and to discriminate from other conditions causing cognitive impairment in prodromal stages. Risk biomarker to predict alterations in cognitively unimpaired individuals (preclinical AD phase).	[89,90]
**TAR DNA-binding protein 43** **(TDP43)**	RNA/DNA-binding protein involved in the regulation of RNA processing.	The accumulation of TDP-43 is a common feature of many neurodegenerative diseases. TDP-43 increases with aging and may act as an age-related mediator of inflammation. Elevated blood levels of TDP-43 have also been found in patients suffering from dementia after cardiac surgery.	[91,92,93]
**Alpha synuclein** **(α-syn)**	Presynaptic protein involved in neurotransmitter release. It has also been identified as a nuclear protein, affecting DNA integrity and transcription	Abnormal alpha synuclein accumulation in neurons form inclusions known as Lewy bodies, the major hallmark of Parkinson’s disease and dementia with Lewy bodies. REM phase can be affected by alpha-synuclein accumulation. The presence of alpha-synuclein in plasma is associated with POD in hip fracture patients.	[94,95,96]
**Neuroinflammation and Immune Response**			
**Interleukins (ILs)**	Inflammatory cytokines	Produced in response to tissue damage and associated with cognitive impairment. As discussed in previous sections, IL-1β, IL-17A, IL-6 and IL-8 could be especially relevant in the connection between surgery and dementia.	See section 2
**Glial fibrillar acid protein (GFAP)/ S100β**	GFAP is involved in the maintenance of the astrocyte structural integrity, whereas S100β is a calcium-binding protein primarily found in astrocytes	GFAP and S100β are reactive astrocytes biomarkers (astrogliosis). Astrocytes can internalize pathological Tau through phagocytosis of dystrophic neurites, an event probably related to astrocyte homeostasis alteration, promoting Tau spreading and contributing to disease progression (early events in AD). Astrogliosis have also been linked to POCD (after cardiac surgery) and POD.	[97,98,99,100]
**Soluble fragment of triggering receptor expressed on myeloid cells 2 (sTREM2)**	The innate immune receptor TREM2 regulates a complex role in microglial metabolism and inflammatory responses	Excessive inflammation via TREM2 may lead to neuronal dysfunction. TREM2 binds to Aβ, triggering a cascade of microglial responses that involve clearance of Aβ toxic aggregates. It is also associated with phosphorylated Tau protein levels and has been recently proposed as a diagnostic biomarker in AD. TREM2 have been shown to functionally interact with ApoE, modulating neuroinflammation and protein clearance. Surgical interventions cause the sTREM2 release, which may participate in the mechanisms that lead to cognitive dysfunction.	[101,102,103,104]
Neurotrophic and Growth Factors			
**Brain-derived neurotrophic factor (BDNF)** **Glial cell line-derived neurotrophic factor (GDNF)**	Neurotrophic factors that play a crucial role in the homeostasis of synaptic plasticity.	BDNF is associated with learning, memory and with the protection of brain in injury-related insults. BDNF deficiency is related to AD, Parkinson or Huntington. Reduced BDNF levels may be associated to cognitive impairment in surgical interventions. Serum GDNF levels are significantly decreased in AD patients, and it has been reported that surgery reduces GDNF levels in the rat brain.	[105,106,107, 108,109]
**Metabolic and Hormonal Regulation**			
**Leptin**, **Uncoupling protein (UCP) and** **Melanocortin 4 receptor (MC4R)**	Leptin is a hormone involved in the regulation of the cellular energy balance, signalling satiety. UCP regulate oxidative stress and synaptic plasticity. MC4R is a G protein-coupled receptor involved in regulating energy homeostasis, appetite, and sexual function.	AD is known to be associated with metabolic anomalies, such as impaired glucose utilization and insulin resistance, which may change heat production regulation. Leptin have been suggested to prevent Tau phosphorylation by the inhibition of GSK-3β. UCP4 variant has been associated to an increased risk of developing AD. The activation of MC4R reduces the Aβ deposition and glial activation in APP/PS1 transgenic AD mouse model.	[110,111,112,113, 114]
**Epigenetic and Regulatory Molecules**			
**Small, non-coding RNAs (miRNAs)**	miRNAs are involved in gene expression regulation and can be transported across the BBB by exosomes	About 250 miRNAs are deregulated in AD patients, with miR-132, miR-425-5p, miR-124-3p or miR-512 being of great relevance in the modulation of tau phosphorylation pathway.	[115]

Several systematic reviews synthesize current evidence on preoperative biomarkers for the prediction of cognitive decline. S100β, neuron-specific enolase, and amyloid beta are promising biomarkers for predicting POCD, for example, but the results of the studies used to draw these conclusions remain limited due to small sample sizes, age restriction, or study heterogeneity [84,85]. The present review brings together a series of useful plasma biomarkers related to inflammation, neuronal damage, or metabolic alterations that could be used to determine the risk of developing dementia or a neurodegenerative disease because of surgery (see table 1). Biomarker monitoring could be of great value to detect a worsening of the patient cognitive status or even to detect a phase transition in the progression of an underlying neurodegenerative disease. The findings from the Global Neurodegeneration Proteomics Consortium may be also useful to search for other biomarkers to detect vulnerable dementia-risk patients [86].

There is common shared biochemical pathway involving most of the biomarkers shown in Table 1.

The relationship leading to a possible neurodegeneration in patients undergoing surgery is shown in Figure 1.

**Figure 1.**
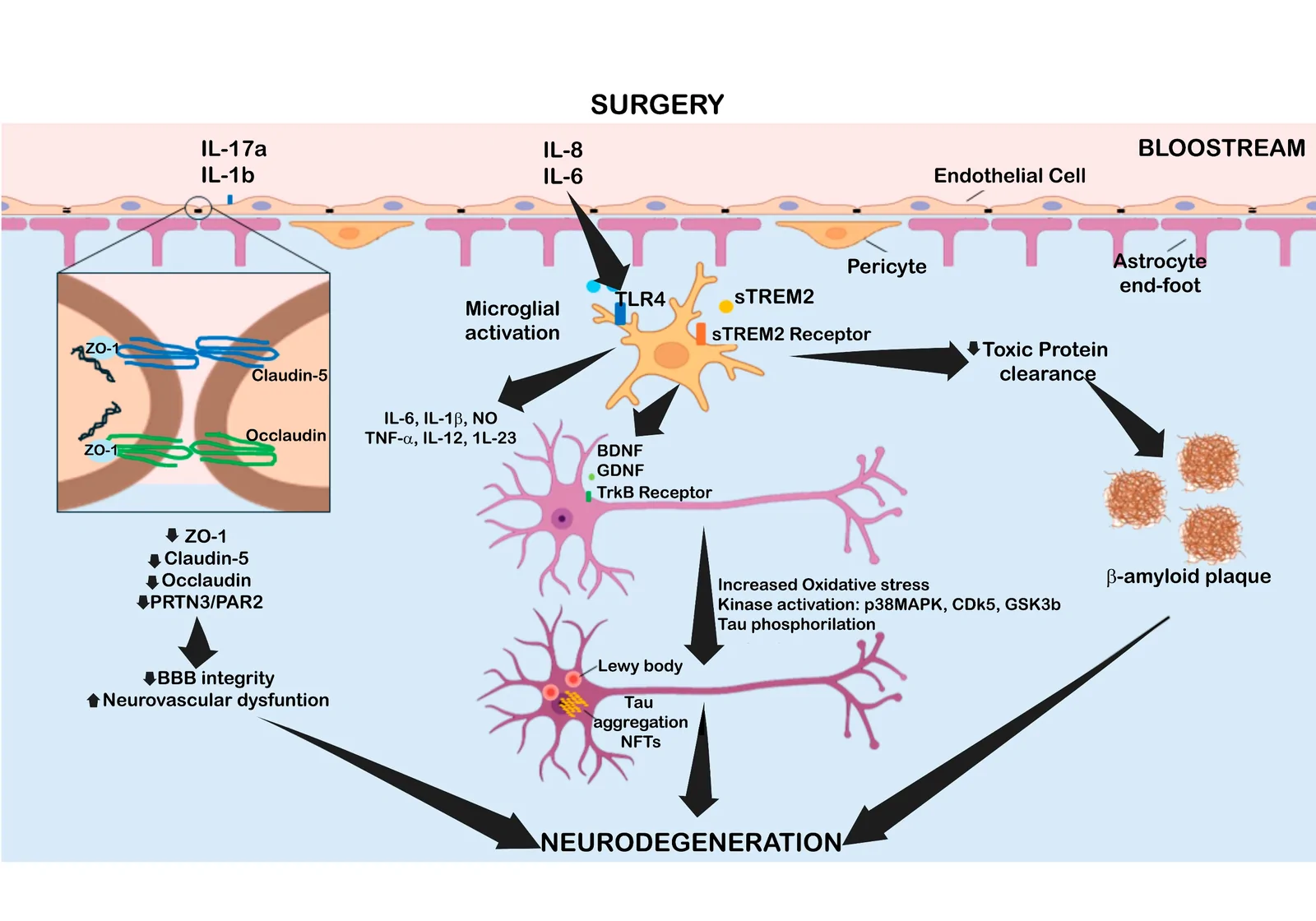
**Role of chronic inflammation in neurodegeneration after surgery**. Cytokine storms affect BBB integrity and promote the M1 microglial phenotype. Exacerbated inflammation may accelerate the aggregation of α-synuclein (Lewy bodies) and influence aggregation and clearance. Proinflammatory microglia amplify the immune response, which will activate kinases involved in Tau phosphorylation: GSK3β (Glycogen Synthase Kinase 3 beta), MAPK (mitogen-activated kinase), and CDK5 (Cyclin-Dependent Kinase 5). At the same time, downregulation of neurotrophic factors such as BDNF and GDNF activates TrkB receptor, enhancing Tau phosphorylation via GSK3β.

In addition to protein/RNA biomarkers, other elements may be considered:

**Blood Brain Barrier (BBB)**: The BBB is a highly selective barrier that communicates the bloodstream and the brain, forming a brain protective shield, mainly composed of endothelial cells, pericytes and astrocytes, interconnected by adhesion molecules [115,116]. Several studies have demonstrated a BBB breakdown in Alzheimer disease, leading to a permeability increase, pericyte degeneration, impaired glucose transport, among others. This may facilitate the entry of neurotoxic substances (Tau cleavage products via exosomes, for example) or external pathogens into the brain [117,118]. Synaptic dysfunction, loss of neuronal connectivity or diminished Aβ clearance are just some examples of the BBB disruption consequences, contributing to the AD pathological process.

The BBB is also destabilized during surgery, becoming more permeable and potentially contributing to POD and POCD [119]. Recent research in mice demonstrated a BBB leakage after laparotomy through an aged-related mechanism, indicating a direct role of surgery/anesthesia that affects the BBB integrity [120].

BBB integrity and permeability may be evaluated using neuroimaging techniques, such as magnetic resonances and PET scans, or with plasma and LCR biomarkers. For example, soluble platelet-derived growth factor receptor beta, which is present on pericytes, has been proposed as a novel biomarker to analyze BBB function [121]. In addition, measuring the levels of tight junction proteins (claudin-5, occludin, ZO-1) or matrix metalloproteinases, may also reflect BBB integrity variations [122,123].

Thus, to evaluate a possible BBB disruption in surgery patients may identify vulnerable patients and act accordingly.

**Gut microbiota**: Recently, gut microbiota dysbiosis has been recognized as a key contributor to the ageing process and is increasingly regarded as a hallmark of ageing. This highlights the microbiome as a critical factor in preserving health and functional capacity throughout the ageing trajectory [124,125]. In this context, international initiatives such as the one led by [126] have called for a revision of the concept of dysbiosis, advocating for an expanded definition that includes not only alterations in microbial composition but also disruptions in microbial function and host-microbiota interactions. These efforts underscore the need for broader, integrative studies exploring the role of the microbiome in health conditions beyond classical gut disorders, including postoperative complications. In relation to this, growing evidence points to the role of dysbiosis in POCD, particularly in older adults. Dysbiosis has been proposed to affect brain function via multiple mechanisms [127,128]. These include modulation of bile acid metabolism [129], promotion of systemic and neuroinflammation, and disruption of neurotransmitter synthesis. Moreover, the microbiota-gut-brain axis is now recognized as an important regulator of glial function and neuroinflammatory processes, particularly involving microglia, which contribute to tau-induced neurotoxicity and cognitive decline [130].

The perioperative context represents a critical window of vulnerability. Commonly used medications such as opioids, anti-inflammatories, and antibiotics can significantly alter gut microbial communities and have been associated with POCD [131]. Recent preclinical studies further support this connection: in aged mice, surgical procedures were shown to induce dysbiosis, increase intestinal permeability, and promote neuroinflammation and cognitive deficits; these effects were mitigated by fecal microbiota transplantation from young donors [132]. Altogether, these findings suggest that perioperative interventions targeting the microbiome may hold promise to prevent or ameliorate cognitive complications associated with surgery, particularly in vulnerable populations such as the elderly.

Finally, the gut microbiota seems to play an active role in regulating body temperature by modulating thermogenesis through brown adipose tissue and the browning of white fat, primarily via microbiota-derived metabolites such as short-chain fatty acids and bile acids that activate sympathetic pathways [133]. However, this remains a largely unexplored area, particularly regarding its implications for surgical interventions, where thermal dysregulation may interact with microbiota to influence recovery and cognitive outcomes.

**Brain activity**: Brain cell function and its patterns of activity have sparked increasing interest as a potentially relevant markers of AD pathology [134] and cognitive function [135]. Brain functional connectivity has been consistently shown to be significantly altered in AD. These alterations exhibit a complex brain synchrony pattern depending on the AD continuum and the specific frequency range [136]. Furthermore, while Aβ42 plaques have been commonly linked to neuronal hyperexcitability, NFTs are known to induce a reduction in synchronization among brain regions [137,138]. Interestingly, an early relationship between brain electrical activity and plasma biomarkers have recently been reported [139,140]: high pTau231 levels correlates with an increase in functional connectivity, or network centrality, between different brain areas [139]. This increase in functional connectivity appears to be related to the subsequent risk of cognitive impairment in patients progressing from mild cognitive impairment to dementia and is considered as an early-stage feature of AD, even in the absence of objectively detectable cognitive impairment [141]. Functionally highly connected regions show similar tau accumulation rates in AD [142], suggesting that Tau spreading occurs through closely interconnected brain regions. Moreover, higher NfL levels in plasma have been recently found to correlate with decreased levels of functional connectivity cross-sectionally, but also with steeper declines in its natural longitudinal evolution [140].

Measuring brain activity before and after surgery could provide critical data to support the presence of other biomarkers in blood and predict cognitive impairment after surgery, particularly since brain cell function has been shown to be highly dependent on spatiotemporal temperature variations [143] and these temperature variations might induce changes in plasma markers closely linked to brain function.

## 7. Patient-level confounders in assessing surgical cognitive risk

The assessment of the effect of anaesthesia or surgery on cognition could be independently influenced by patient-related biases. Factors inherent to the patient, such as lifestyle (diet, physical activity, alcohol, nicotine or other substances consumption), frailty or mood disorders may confound the assessment of surgical cognitive risk. In addition, pre-existing medical conditions (such as hypertension or diabetes) may influence cognitive outcomes after surgery or create a complex landscape of connections between measured biomarkers that require careful adjustment to avoid misinterpretation. In this regard, study design should adjust potential confounders separately for each risk factor to ensure more reliable results [144].

## Conclusions and future perspectives

Several surgical factors have been proposed as potential triggers of mental derangements in elderly patients, and a battery of biomarkers has been postulated for patient state screening (table 1). Most of the biomarkers from table 1 are not routinely measured in hospitals, but they may contribute to the detection of at-risk populations vulnerable to a side-effect of anesthesia and prevent the development of cognitive deficits, if specific treatment is administrated, which is crucial in the current health care system. Thus, clinical practice guidelines can be improved by implementing new approaches in perioperative and post-operative protocols. Through this review, we recommend monitoring body temperature and preventing drops during surgery to reduce surgical complications and speed up patient recovery. Post-surgical interventions can also be improved, for example, by specific nutritional strategies to address imbalances in the gut microbiota, especially after antibiotics administration. This could have a beneficial effect on the microbiota-gut-brain axis in the long term. On the other hand, it would also be important to improve sleep quality, as this would allow for better restoration of brain function (by optimizing sleep-wake cycles or through a pharmacological approach). Finally, constant monitoring of biomarkers related to neuroinflammation or neurodegeneration could be helpful in making decisions about long-term pharmacological treatments.

Further prospective and longitudinal studies will elucidate the value of these biomarkers and will help to better understand the mechanisms underlying surgical cognitive declines, such as POD or POCD, and associated neurodegenerative diseases. In relation to this, the brain temperature drop, and the phosphorylation of Tau protein emerges as a relevant research field (see Fig. 2).

**Figure 2.**
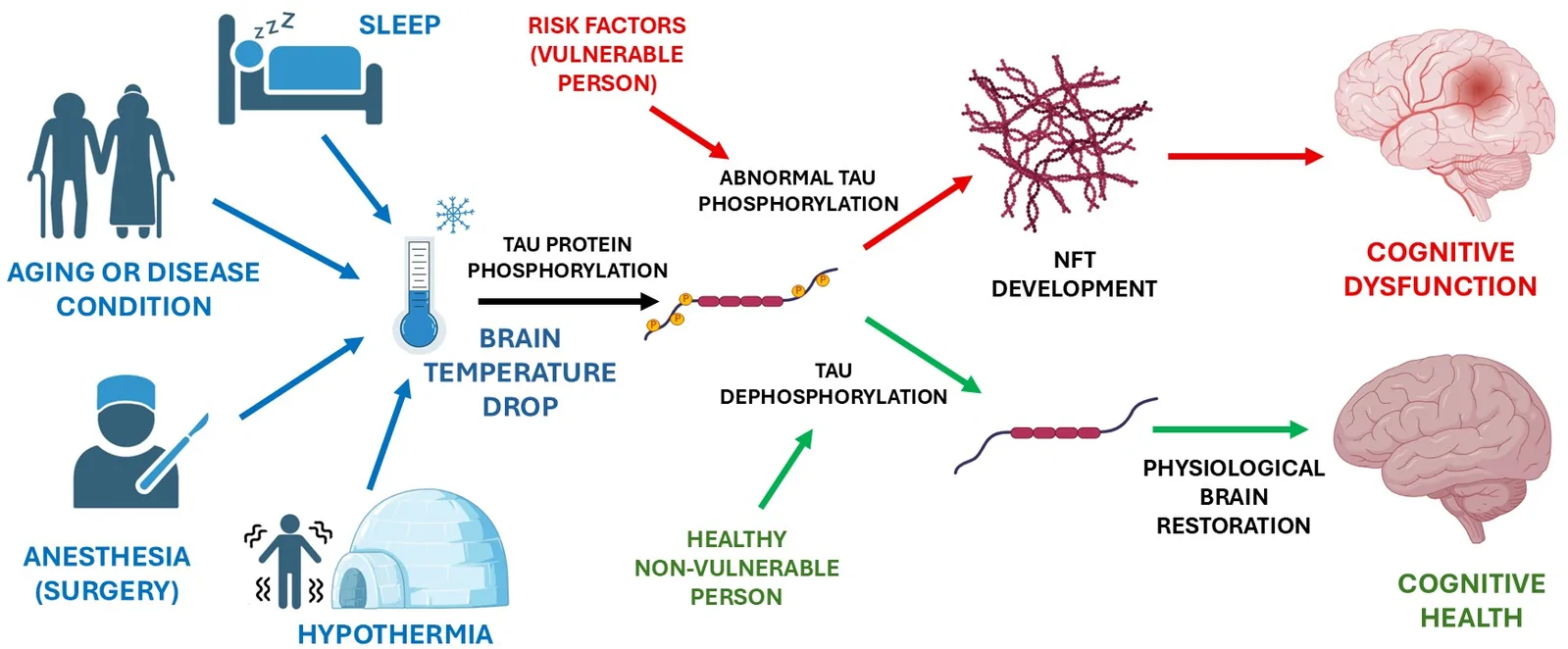
**Schematical image showing the relationship between Tau hyperphosphorylation, NFT neuronal formation and cognitive dysfunction**. All processes share common mechanisms involving brain temperature decrease.
